# Multi-mode joint modulation of array wireless power transfer

**DOI:** 10.1038/s41598-023-42983-5

**Published:** 2023-09-22

**Authors:** Da Li, Xusheng Wu, Wei Gao, Jianxin Gao

**Affiliations:** 1 School of Electrical Engineering, Naval University of Engineering, Wuhan, China; 2 National Key Laboratory of Electromagnetic Energy, Naval University of Engineering, Wuhan, China

**Keywords:** Electrical and electronic engineering, Energy infrastructure

## Abstract

In this paper, an array multi-transmitter multi-mode wireless power transfer system is proposed. The system realizes the joint modulation of three transmitter coil working modes of single transmitter coil, dual transmittercoil and four transmitter coils in the wireless power transfer system through PI closed-loop control, which can realize the stable output of WPT system in three different transmitter coil work modes. It effectively compensates for the shortcomings of the single working mode of a single transmitter coil and the limited range of effective working areas, and improves the robustness of the wireless power transfer system.

## Introduction

Limited by the power density of the UAV battery, the cruising range of the UAV is limited. The wireless power transfer technology can power the onboard battery without physical connection, thus eliminating manual intervention and realizing automatic charging of the UAV, and expanding the scope of work. In the wireless power transfer system, due to the random change of the position of the receiving coil, the center of the receiving coil and the transmitting coil is misplaced. The increase of the offset between the transmitting coil and the receiving coil in the WPT system will reduce the coupling coefficient between the transmitting coil and the receiving coil, resulting in the WPT system unable to achieve stable output^1^.

In the single-transmitter single-receiver wireless power transfer system, the power efficiency characteristics of the WPT system are affected by the circuit load, the coupling coefficient between the coils, and the coil quality factor^2,3^. The power efficiency characteristics of the WPT system can be effectively improved by optimizing the coil design and compensating the network parameters to achieve impedance matching to obtain the best load and automatically adjust the operating frequency^4,5^. The significant change in the relative position between the transmitter and receiver coil will decrease the coupling coefficient between the transmitter and receiver coil, which leads to the limited power and efficiency optimization of the WPT system. The multi-transmitter wireless power transfer system can increase the working area of the wireless power transfer system and change the anti-offset performance of the WPT system under the offset of the transmitter coil and the receiver coil, so it has become a research hotspot of wireless power transfer^6–8^.

According to^9^, the field-oriented transfer between the transmitter and the receiver coils is realized by a multi-transmitter coil composed of a hollow coil matrix, effectively reducing the power loss. However, there is a weak coupling at the edge of the adjacent transmitter coil, resulting in a sharp decline in the transmission efficiency of the wireless power transfer system and the power along the boundary of the transmitter coil. According to^10^, the wireless power transfer of the receiver coil in three-dimensional space is realized by optimizing the current size and phase of the cross-overlap array transmitter coil. According to^11^, a wireless power transfer system for mobile phones is proposed to transmit power to multiple loads at any position in the charging area. Due to the position uncertainty of the receiver coil, the coupling coefficient between the transmitter coil and the receiver coil fluctuates greatly, which affects the power efficiency characteristics of the WPT system. The control switching method of the existing array wireless power transfer system is switching a single transmitter coil, but there is a power and efficiency drop at the boundary of the transmitter coil. The simultaneous operation of multiple inverters and multiple transmitter coils will cause the magnetic circuit of the WPT system to be too open, resulting in a significant magnetic field leakage. Therefore, for the array transmitter, the segmented control method can be used to achieve the work of the target coil and reduce the magnetic flux leakage of the array transmitter coil. Based on the multi-transmitter wireless power transfer system, D. Ahn used a fully activated multilayer planar transmitter coil to increase the working area of the WPT system, but it caused unnecessary magnetic field leakage and power loss^12^. The research team of the Swiss Federal Institute of Technology in Lausanne proposed a 3 × 3 square coil multi-transmitter array to supply power to loads such as mouse and keyboard. However, the double-layer structure of the transmitter coil is complex, and the adaptability of the load and charging is not strong when the load is rotated^13^. The research team of the University of Zaragoza in Spain proposed a power supply plane with a staggered arrangement of transmitter coils. It proposed a parallel connection method for coil connection and control. This coil connection method reduces the amount of switching and reduces the switching loss, but it requires an additional detection circuit, which in turn increases the complexity of the system^14^. The team also designed and implemented a multi-transmitter coil structure with a keyboard array. By controlling the switch tubes corresponding to different rows and columns, the switching control of the on-state of the transmitter coil is realized^15^. However, this connection and control structure cannot control each transmitter coil separately.

The optimization of UAV batteries is difficult to achieve a significant improvement in UAV endurance. Frequent battery replacement will significantly reduce the utilization rate and convenience of UAV. The autonomous power replenishment of UAV through wireless power transfer technology can effectively expand their flight radius, which is a key link in achieving intelligent systems. The research team at Imperial College of Technology in the UK has integrated the drone's collision avoidance frame with the receiver coil, minimizing changes to the drone's appearance and payload. However, due to the fact that the receiver coil of the drone is at the same level as the UAV body, a large amount of magnetic flux will pass through the drone, causing electromagnetic interference to its internal components and circuits, and affecting the stability of the UAV operation^16^. The Korean Academy of Science and Technology adopts a single transmitter single receiver coupler, and placing the receiving coil directly below the drone will generate electromagnetic interference, affecting the operation of other electronic components on the drone^17^. The University of L'Aquila in Italy adopts a multi emission coil magnetic coupling structure, which not only reduces the interference of high-frequency magnetic fields on drone electrical equipment, but also expands the charging area of drones and improves their anti drift performance^18^. Kyushu University in Japan adopts capacitive power transmission technology to achieve sufficient power for drones in a short period of time. However, this device requires complex matching circuits and low transmission power^19^. The University of Trento in Italy adopts magnetic resonance WPT technology to achieve unmanned aerial vehicles (UAVs) suspended above the transmission coil in a state without batteries^20^. However, this type of transfer coil structure requires multiple transmitter coils to work together, making it difficult to design and control. Due to factors such as insufficient landing accuracy and environmental interference, the relative misalignment between the receiver coil and the transmitter coil is a problem that UAV must solve to achieve efficient and stable wireless power transfer.

The array wireless power transfer system currently adopts single transmitter coil switching to realize wireless power transfer. However, the array transmitter coil cannot achieve flexible regional control, and the transmitter side circuit needs to remain open during the operation of the WPT system, resulting in magnetic field leakage and reduced power and efficiency. In this paper, the work modes of the three transmitter coils of the WPT system are designed, and the combined modulation of the work modes of the multitransmitter coils is realized based on the PI closed-loop control to realize the stable output of the WPT system. For the receiver voltage at the load end of the array multi-transmitter WPT system, it can be provided by three transmitter coil working modes : single transmitter coil, dual transmitter coil and four transmitter coil. That is, the output of a certain state of the WPT system can be realized by three transmitter coil working modes.

## Array multi-transmitter wireless power transfer system

### Multi-transmitter coil structure

The cross-coupling between the traditional array transmitter coils will consume the transfer power of the transmitter coil, affect the impedance calculation between the transmitter coils, and increase the complexity of modeling and calculation of the wireless power transfer system^21,22^. The BP (Bipolar pad) and DD (Double D) coils minimize cross-coupling between the transmitter coils by overlapping the transmitter coils^23^. Therefore, this paper realizes the decoupling between adjacent transmitter coils by overlapping between adjacent transmitter coils^24^, and the mutual inductance between non-adjacent transmitter coils can be ignored^25^.

**Figure 1:**
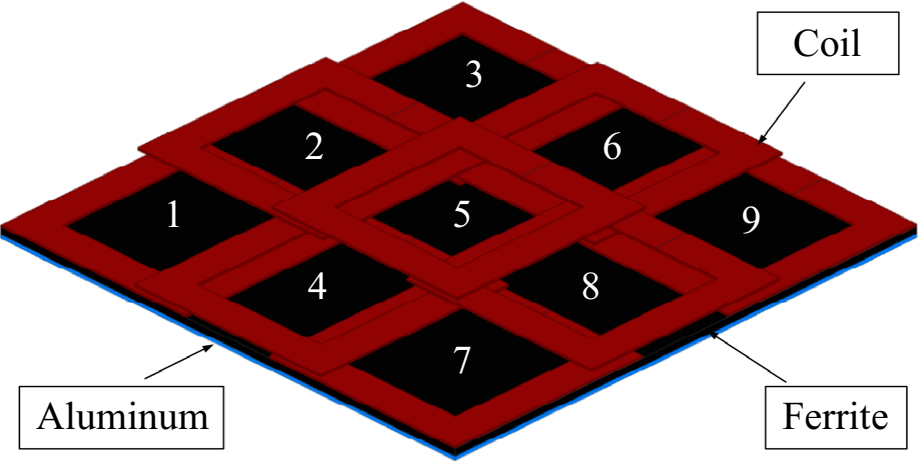
Heterophane overlapping array transmitter coil.

The multi transmitter coil designed in this article with different overlapping arrays is shown in Fig. 1. The overlapping area between two adjacent transmitter coils is adjusted to realize the decoupling of adjacent transmitter coils. The overlap distance between two adjacent coils is adjusted so that the size and direction of the transmitter coil penetrating flux $\psi_{1}$ and penetrating flux $\psi_{2}$ are equal and opposite, and the mutual decoupling between two adjacent transmitter coil units can be realized. The structure and coupling relationship of overlapping array transmitters is very complex. For multi-turn coils with complex three-dimensional structures, it will not be easy to obtain results if the Maxwell equation is used for calculation. However, the Maxwell equations can significantly improve the efficiency of design. To approximately solve the mutual inductance between two overlapping coils, it is simplified as a single turn wire circuit, and the mutual inductance value between the two coils is approximated using the Maxwell equations.

1 $$ \begin{gathered} M(r_{i} ,r_{j} ,L,D) = \mu \pi \sqrt {r_{i} \cdot r_{j} } \int\limits_{0}^{\infty } {J_{1} \left(x\sqrt {\frac{{r_{i} }}{{r_{j} }}} \right)} J_{1} \left(x\sqrt {\frac{{r_{j} }}{{r_{i} }}} \right) \cdot \, \hfill \\ \, J_{0} \left(\frac{xL}{{\sqrt {r_{i} r_{j} } }}\right)g \times \exp \left( - x\frac{D}{{\sqrt {r_{i} r_{j} } }}\right)dx \hfill \\ \end{gathered} $$

where $\mu$ is the permeability, $D$ is the vertical coupling distance between two wire loops, $L$ is the transverse distance between the centres of two wire loops, $J_{0}$,$J_{1}$ are the Bessel function of order 0 and 1. $r_{i}$,$r_{j}$ are half of the side length of the coils *I* and *J*, respectively. The multi-turn transmitter coil can be treated approximately as each turn coil. The mutual inductance value between multi-turn coils can be obtained.

2 $$ M_{ij} = \sum\limits_{i = 1}^{{N_{i} }} {\sum\limits_{j = 1}^{{N_{j} }} {M(r_{i} ,r_{j} ,L,D)} } $$

where $N_{i}$,$N_{j}$ are turns of coil *I* and coil *J*, respectively. Since the mutual inductance between non-adjacent coils can be ignored.

**Figure 2:**
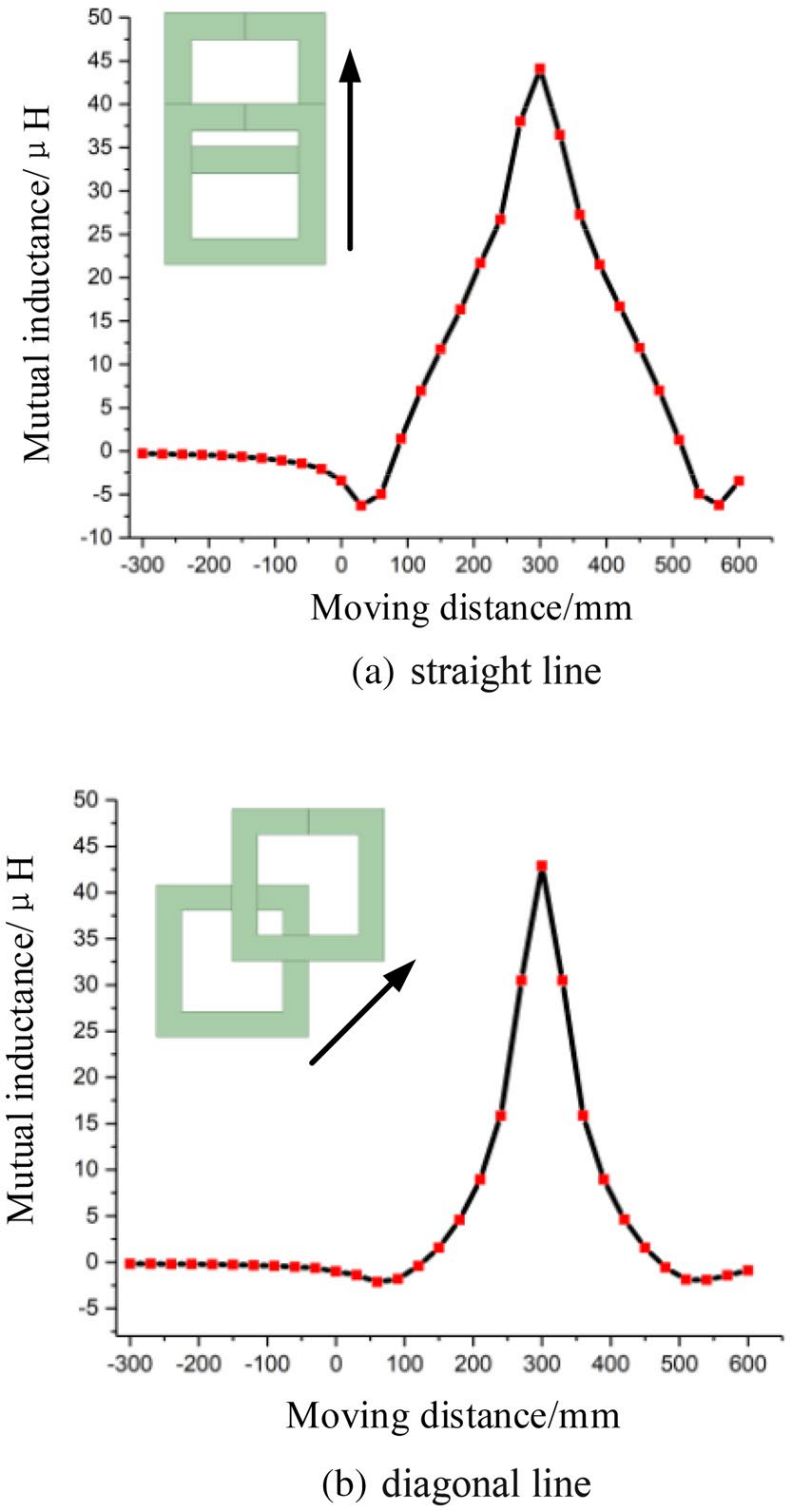
Mutual inductance and overlap distance between coils.

The transmitter model of 300 mm × 300 mm × 5 mm is built in Maxwell, and the coil is moved along the side of the coil and diagonally to obtain the mutual inductance between the two coils at different overlapping positions in Fig. 2. The mutual inductance between the transmitter coil and the receiver coil is 28.75–34.56 μH. The maximum mutual inductance of the two transmitter coils is 1.42 μH. The maximum mutual inductance value between transmitter coils is less than 5% of the mutual inductance value between transmitter and receiver coils. It can be considered that each unit of the array multi-transmitter coil can work independently.

### Coil grouping and control logic

**Figure 3:**
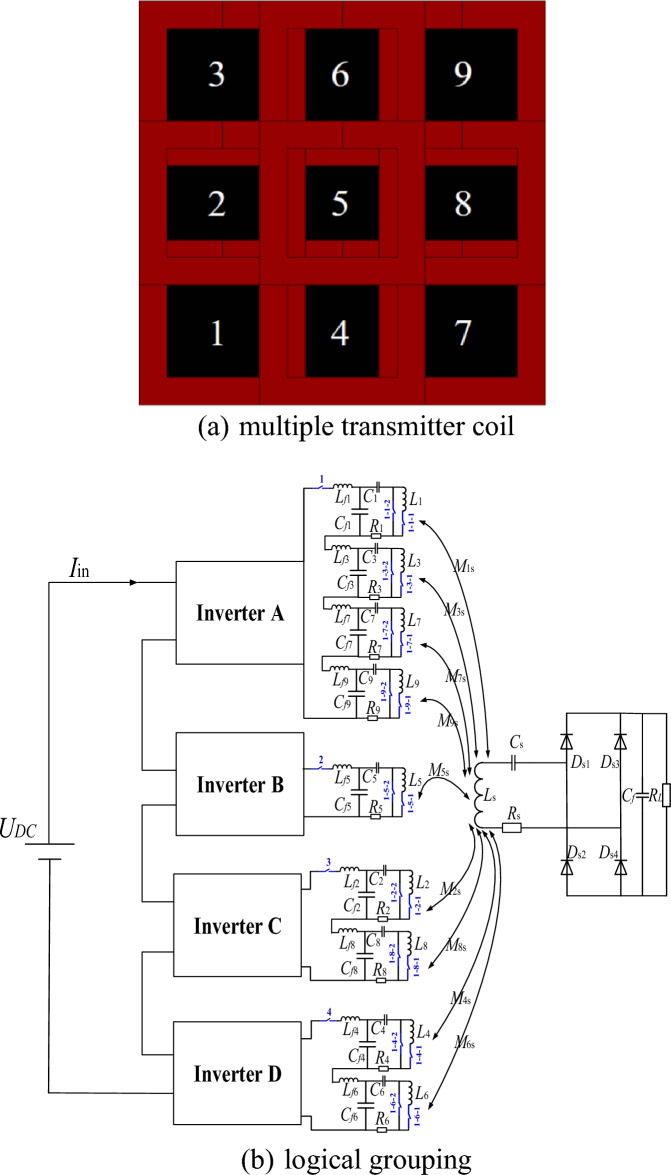
Structure diagram of array multi-transmitter system.

The simultaneous operation of array multi-transmitter coils will lead to the large magnetic field leakage. The traditional array multi-transmitter coil adopts the control mode of a single inverter-single transmitter coil, which has low transfer power, weak anti-interference ability, and cannot fully use the cooperative work of multiple transmitter coils to realize wireless power transfer. This paper proposes a grouping logic and control strategy for array multi-transmitter coils. Through the short-circuit crossover switch, and the open-circuit series switch, the cooperative power supply of any 1-TX coil, any adjacent 2-TX coils, and any adjacent 4-TX coils can be realized without changing the coil topology. The structure diagram of the array multi-transmitter WPT system is shown in Fig. 3. To achieve conflict-free optimal control. Four inverters control nine transmitter coils, and any adjacent 4-TX coils are powered simultaneously. The switching control of different working modes of the array transmitter coil is realized by four inverters. The grouping logic of multiple transmitter coils is shown in Fig. 3. All switching components are AC contactors CJX2-1810. The transmitting coils 1, 3, 7 and 9 are located at the corners of the array transmitter coil respectively, and the electromagnetic interference between them is small, so the inverter A is selected to drive. The transmitter coil 2, 6 and the transmitter coil 4, 8 are respectively located at the diagonal of the transmitter coil, which can be driven by the same inverter C and D. The transmitter coil 5 is located at the center of the array multi-transmitter coil and works in any four-transmitter coil work mode, so a separate inverter B is selected.

### Output performance analysis of multi-transmitter wireless power transfer system

**Figure 4:**
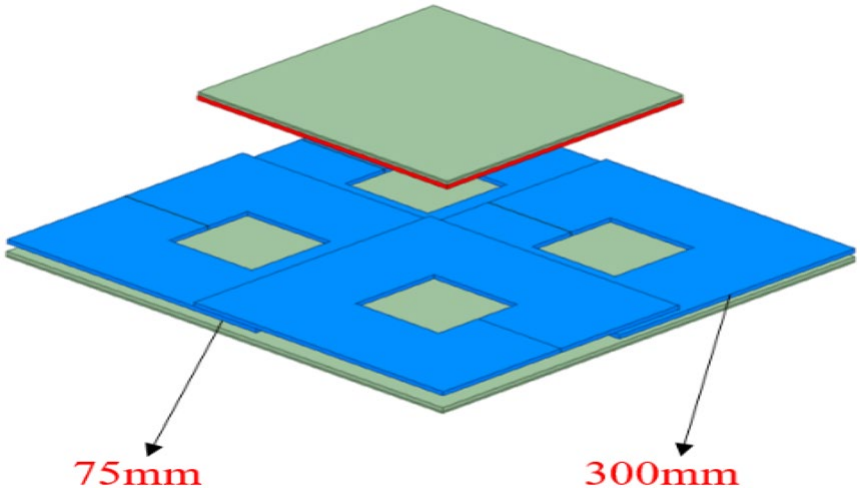
Structure diagram of array multi-transmitter system.

The four-transmitter coil working mode of the 3 × 3 array wireless power transfer system described in this paper can include three working modes: single transmitter, dual transmitter coil, and four transmitter coils. Therefore, the output performance analysis of the array wireless power transfer system is illustrated by taking the four transmitter coils as an example. In Fig. 4, the size of the transmitter coil is 300 mm × 300 mm × 5 mm, and the overlap distance between the transmitter coils is 75 mm. According to the above analysis, the mutual inductance between the transmitter coils can be ignored at this overlap distance, which is conducive to the independent operation of multiple transmitter coils. The receiver coil is in the middle part of the array transmitter coil, and the vertical distance between the receiver coil and each transmitter coil is the same. That is, $M_{1s} = M_{2s} = M_{3s} { = }M_{4s}$.

**Figure 5:**
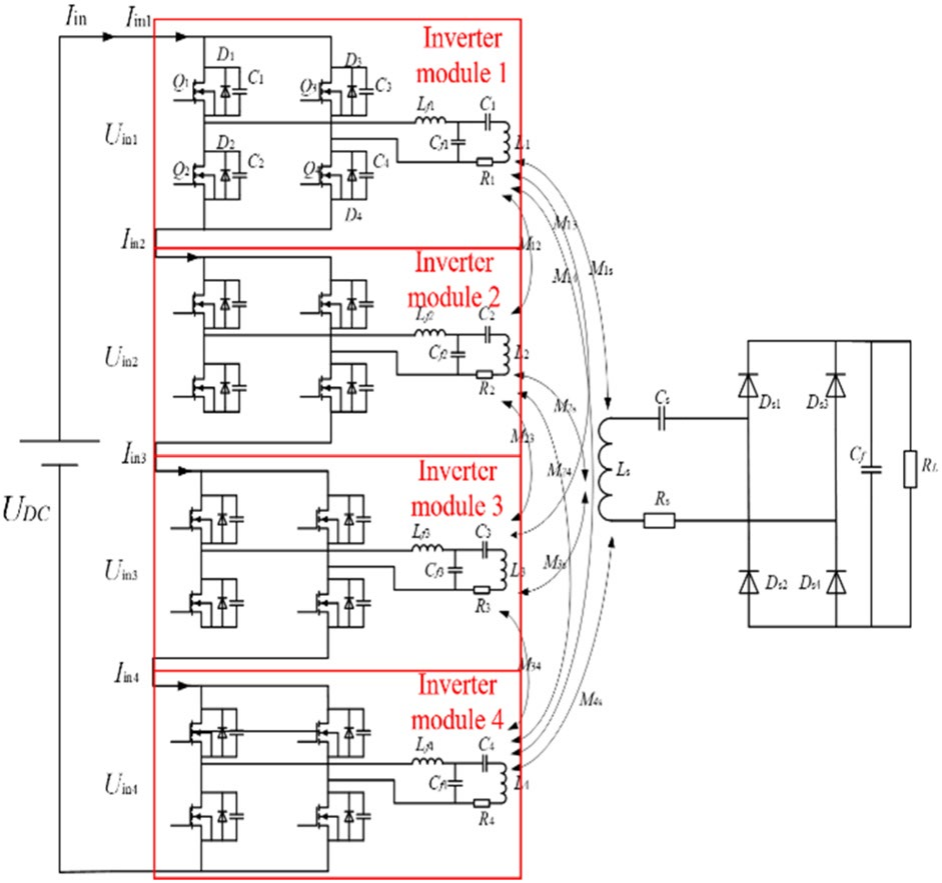
Topology of four-transmitter single-receiver WPT system.

The topology of the four-transmitter single-receiver WPT system based on four channels is shown in Fig. 5. Each branch of the array multi-transmitter WPT system can work independently. The four inverter channels are all powered by the DC power supply *U*_DC_, and the four inverter channels adopt a series input structure.

The relationship between the input voltage of each inverter channel and the DC source voltage can be obtained:

3 $$ U_{DC} { = }U_{in1} + U_{in2} + U_{in3} + U_{in4} $$

The four-branch inverter and the compensation network parameters are the same under ideal conditions:

4 $$ U_{in1} { = }U_{in2} { = }U_{in3} { = }U_{in4} $$

It can be seen that the output voltage of the four inverter channels is the same, and the high voltage output of the multi-channel inverter power supply can be effectively realized by the simultaneous output of the four inverter channels. The four-transmitter single-receiver WPT system is in a steady-state working state:

5 $$ I_{in} = I_{in1} = I_{in2} = I_{in3} = I_{in4} $$

*M*_xy_ refers to the mutual inductance between the coils, $I_{o1} ,I_{o2} ,I_{o3} ,I_{o4}$ are the output current of four channels in the WPT system. The induced voltage in the receiver is:

6 $$ U_{0} = j\omega MI_{1s} I_{01} + j\omega MI_{2s} I_{02} + j\omega MI_{3s} I_{03} + j\omega MI_{4s} I_{04} $$

According to Eq. (6), the induced voltage of the receiver coil is related to the mutual inductance between the transmitter and receiver coils, and the mutual inductance between the transmitter and receiver coils is related to the arrangement of the transmitter coils. The arrangement of the four transmitter coils in the multi-transmitter WPT system is shown in Fig. 4. It can be obtained:

7 $$ U_{0} = j\omega Mp_{0} (I_{01} + I_{02} + I_{03} + I_{04} ) $$

The four transmitter coils of the multi-transmitter WPT system work simultaneously, according to Kirchhoff law:

8 $$ \left\{ \begin{gathered} u_{1} = j\omega Li_{in1} + (j\omega L_{p} + \frac{1}{{j\omega C_{p} }} + r_{p1} )i_{p1} + j\omega M_{s1} i_{s} \hfill \\ u_{2} = j\omega Li_{in2} + (j\omega L_{p} + \frac{1}{{j\omega C_{p} }} + r_{p2} )i_{p2} + j\omega M_{s2} i_{s} \hfill \\ u_{3} = j\omega Li_{in3} + (j\omega L_{p} + \frac{1}{{j\omega C_{p} }} + r_{p3} )i_{p3} + j\omega M_{s3} i_{s} \hfill \\ u_{4} = j\omega Li_{in4} + (j\omega L_{p} + \frac{1}{{j\omega C_{p} }} + r_{p4} )i_{p4} + j\omega M_{s4} i_{s} \hfill \\ 0 = j\omega M_{s1} i_{p1} + j\omega M_{s2} i_{p2} + j\omega M_{s3} i_{p3} + j\omega M_{s4} i_{p4} { + }i_{s} ({\text{R}}_{eq} + r_{s} ) \hfill \\ \end{gathered} \right. $$

$R_{eq} = 8R/\pi^{2}$ is the equivalent resistance after the rectification circuit. The input current and load current of each resonant network can be obtained as follows:

9 $$ \left\{ \begin{gathered} i_{in1} = \frac{{u_{ab} (\omega^{2} M_{s1} (M_{s1} + M_{s2} { + }M_{s3} + M_{s4} ) + (r_{s} + {\text{R}}_{eq} )r_{p} )}}{{(r_{s} + {\text{R}}_{eq} )\omega^{2} L^{2} }} \hfill \\ i_{in2} = \frac{{u_{ab} (\omega^{2} M_{s2} (M_{s1} + M_{s2} { + }M_{s3} + M_{s4} ) + (r_{s} + {\text{R}}_{eq} )r_{p} )}}{{(r_{s} + {\text{R}}_{eq} )\omega^{2} L^{2} }} \hfill \\ i_{in3} = \frac{{u_{ab} (\omega^{2} M_{s3} (M_{s1} + M_{s2} { + }M_{s3} + M_{s4} ) + (r_{s} + {\text{R}}_{eq} )r_{p} )}}{{(r_{s} + {\text{R}}_{eq} )\omega^{2} L^{2} }} \hfill \\ i_{in4} = \frac{{u_{ab} (\omega^{2} M_{s4} (M_{s1} + M_{s2} { + }M_{s3} + M_{s4} ) + (r_{s} + {\text{R}}_{eq} )r_{p} )}}{{(r_{s} + {\text{R}}_{eq} )\omega^{2} L^{2} }} \hfill \\ i_{s} = - \frac{{j\omega (M_{s1} + M_{s2} { + }M_{s3} + M_{s4} )i_{p} }}{{{\text{R}}_{eq} + r_{s} }} \hfill \\ \end{gathered} \right. $$

The primary side current of the WPT system is related to the mutual inductance between the transmitter and receiver coils. The mutual inductance of the receiver coil and the four transmitter coils is equal, and the output current and output power of the four inverter channels are equal. It can be obtained that the input impedance of the WPT system under the working mode of the dual transmitter coil and the working mode of the four transmitter coils are:

10 $$ \left\{ \begin{gathered} Z_{2} = \frac{{\omega^{2} L^{2} }}{{\frac{{2\omega^{2} M^{2} }}{{(R_{eq} + r_{s} )}} + r_{p} }} \hfill \\ Z_{4} = \frac{{\omega^{2} L^{2} }}{{\frac{{4\omega^{2} M^{2} }}{{(R_{eq} + r_{s} )}} + r_{p} }} \hfill \\ \end{gathered} \right. $$

The mutual inductance between the receiver coil and the four transmitter coils is equal, and the reflected impedance of the WPT system under the working mode of the four transmitter coils and the working mode of the dual transmitter coils is four times and two times that of the WPT system under the single transmitter coil mode, respectively. It can be obtained that the output voltage of the WPT system in these two transmitter coil operating modes is:

11 $$ \left\{ \begin{gathered} u_{o2} = 2\omega Mi_{p} = \frac{{4u_{ab} M}}{L} \hfill \\ u_{o4} = 4\omega Mi_{p} = \frac{{4u_{ab} M}}{L} \hfill \\ \end{gathered} \right. $$

And the output power of the WPT system in the dual transmitter coil and the four transmitter coil is:

12 $$ \left\{ \begin{gathered} P_{{out{ - }2}} = \frac{{4\omega^{2} M^{2} i_{p}^{2} R_{eq} }}{{(R_{eq} + r_{s} )^{2} }} = \frac{{4M^{2} u_{ab}^{2} R_{eq} }}{{(R_{eq} + r_{s} )^{2} L^{2} }} \hfill \\ P_{{out{ - }4}} = \frac{{16\omega^{2} M^{2} i_{p}^{2} R_{eq} }}{{(R_{eq} + r_{s} )^{2} }} = \frac{{16M^{2} u_{ab}^{2} R_{eq} }}{{(R_{eq} + r_{s} )^{2} L^{2} }} \hfill \\ \end{gathered} \right. $$

Compared with the WPT system in the single transmitter coil, the output current and voltage of the WPT system in the 2-TX coil are increased by two times, and the output power is increased by four times. Compared with the WPT system in the 1-TX coil, the output current and voltage of the WPT system in the four transmitter coil are increased by four times, and the output power is increased by 16 times. The LCC-S compensation network has the characteristics of a constant current of the transmitter coil. The current of the transmitter coil is independent of the mutual inductance between the transmitter and receiver coil and the system's load. The reflection impedance of the WPT system in the 2-TX is twice of the WPT system in the 1-TX coil. The current of the transmitter coil in the dual transmitter coils is equal, and the input current of the resonant network in the dual-transmitter coil is close to twice the WPT system in the 1-TX coil. The reflection impedance of the WPT system in the 4-TX coil is four times that of the WPT system in the 1-TX coil, and the current of the resonant network is approximately four times that of the WPT system in the 1-TX coil.

**Figure 6:**
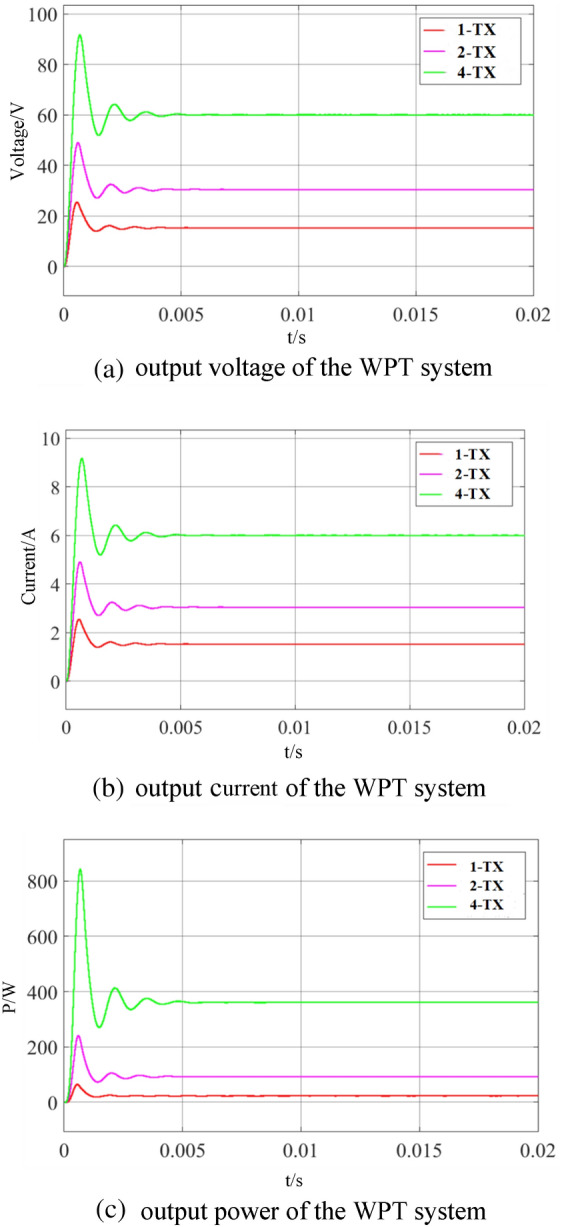
WPT System output performance comparison.

Figure 6 is the simulation curve of the output performance of the WPT system under the 4-TX coil mode, the 2-TX coil mode and the 1-TX coil mode. The output voltage and current of the WPT system under the 2-TX coil mode are 2 times that of the WPT system under the 1-TX coil mode, and the output power is 4 times that of the WPT system under the 1-TX coil mode. The output voltage and current of the WPT system in the 4-TX coil mode are 4 times that of the WPT system in the 1-TX coil mode, and the output power is 16 times that of the WPT system in the 1-TX coil mode.

**Figure 7:**
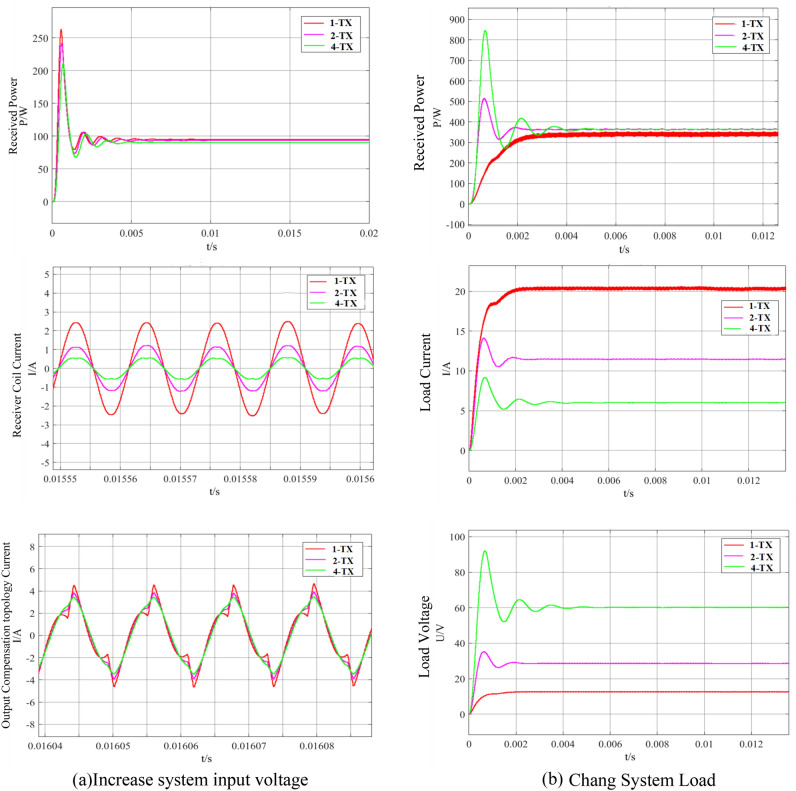
Simulation waveform of WPT system at the same output power.

The transer distance and coil parameters of the WPT system are determined, the output power of the WPT system can be changed by adjusting the input voltage or system load of the WPT system. Through the simulation curve of Fig. 7, it can be seen that increasing the input voltage of the WPT system, the output power of the WPT system with 1-TX mode is the same as that of the WPT system with 4-TX mode. In the1-TX mode, the current of the transmitter coil of the WPT system increases by 4 times, and the voltage stress of the switching device increases by 4 times. The increase of the transmitter coil current leads to the increase of the WPT system. When the load of the WPT system in the 1-TX coil mode is reduced by 16 times, the output power of the WPT system in the 4-TX mode is the same. The input voltage is the same, so the current of the transmitter coil in the two WPT systems is the same. The primary equivalent impedance of the WPT system in the 1-TX mode is only 1/4 of that in the 4-TX mode. Therefore, the input current of the compensation network of the WPT system in the 1-TX mode is 4 times that of the WPT system in the 4-TX mode, which greatly increases the current stress of the switching devices. Therefore, when the output power of the WPT system in the 4-TX mode is the same as that of the WPT system in the 1-TX mode, the voltage and current stress of the WPT system device in the 4-TX mode is only 1/4 of that of the WPT system in the 1-TX mode, which effectively reduces the requirements of the WPT system for device performance.

### Analysis of closed-loop control system based on LCC-S compensation network

The coupling mutual inductance between the transmitter and receiver coils in the 4-TX coil WPT system is easy to change, and there are multiple linear environments such as high-frequency inverters, resonance compensation networks, and rectification filtering in the WPT system. Therefore, the Generalized State Space Averaging (GSSA) is used to establish the generalized state-space equation of the WPT system. The 4-TX coil is used to realize the decoupling between adjacent transmitter coils through different surface superpositions, so the one channel in the multi-transmitter WPT system can be simplified and analyzed.

**Figure 8:**
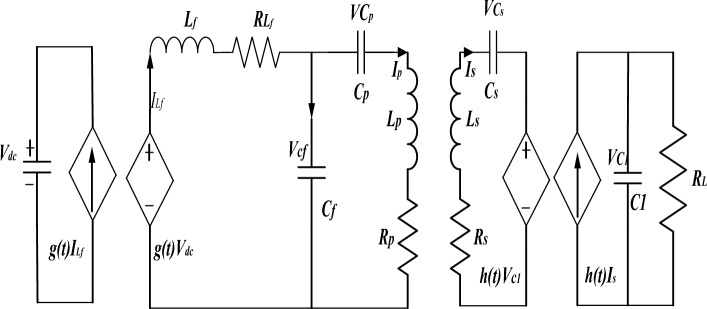
Equivalent circuit diagram of LCC-S type WPT system.

**Figure 9:**
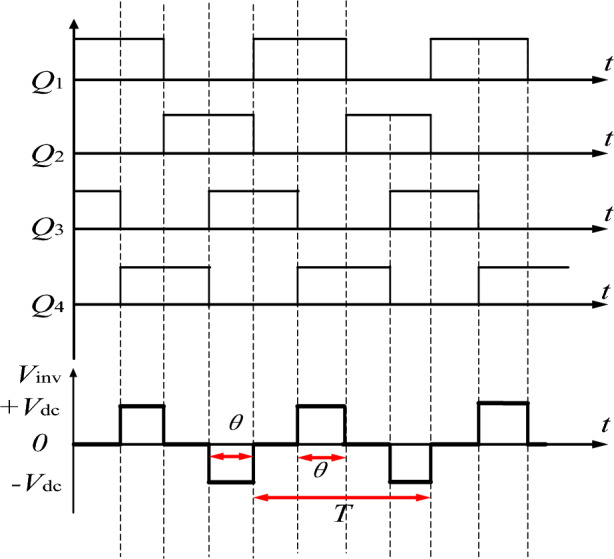
Relation curve between phase shift angle and inverter output voltage.

The equivalent circuit of the WPT system based on the LCC-S compensation network is shown in Fig. 8. Figure 9 shows the waveform of the phase shift angle and the output voltage of the inverter circuit in the full-bridge inverter circuit. The switch tube $Q_{1}$ and $Q_{2}$ are complementary, and the switch tube $Q_{3}$ and $Q_{4}$ are complementary. The operating frequency of the switch in the inverter circuit is $f_{inv}$, the cycle is $T$, and the forward conduction time and reverse conduction time of the inverter circuit are $\theta /2\pi f_{inv} = (\theta /2\pi )T$,$0^{ \circ } \le \theta \le 180^{ \circ }$.

The power conversion links, such as the primary high-frequency inverter and secondary side rectifier in the WPT system, have nonlinear characteristics. Therefore, the primary side inverter full-bridge power conversion function is defined as *g(t)*, and the rectifier power conversion function is defined as *h(t)*. The inverter output voltage is:

13 $$ V_{inv} = g(t)V_{dc} = \left\{ \begin{gathered} 0 \, t \in \left[ {nT,\left( {n + \frac{\pi - \theta }{{2\pi }}} \right)T} \right] \hfill \\ 1 \, t \in \left[ {\left( {n + \frac{\pi - \theta }{{2\pi }}} \right)T,\left( {n + \frac{1}{2}} \right)T} \right] \hfill \\ 0 \, t \in \left[ {\left( {n + \frac{1}{2}} \right)T,\left( {n + \frac{1}{2} + \frac{\pi - \theta }{{2\pi }}} \right)T} \right] \hfill \\ 1 \, t \in \left[ {\left( {n + \frac{1}{2} + \frac{\pi - \theta }{{2\pi }}} \right)T,(n + 1)T} \right] \hfill \\ \end{gathered} \right.\quad n \in N $$

Put the Fourier series expansion of inverter output voltage (Eq. 13), and take its fundamental practical value as:

14 $$ V_{inv} = \frac{2\sqrt 2 }{\pi }V_{dc} \sin \left( {\frac{\theta }{2}} \right) $$

*n* is an integer multiple of the switching period *T* of the inverter. The full bridge circuit of the inverter injects power into the forward and reverse resonant topology of the primary side using *g*(*t*) logical values 1 and −1, respectively. The logic values used by the secondary side rectifier bridge for forward and reverse rectification of received electrical power *h*(*t*) are 1 and −1.

15 $$ h(t) = \left\{ \begin{gathered} 1 \, t \in \left[ {\left( {n + \frac{\varphi }{2\pi }} \right)T,\left( {n + \frac{\varphi }{2\pi }{ + }\frac{1}{2}} \right)T} \right] \hfill \\ { - }1 \, t \in \left[ {\left( {n + \frac{\varphi }{2\pi }{ + }\frac{1}{2}} \right)T,\left( {n + \frac{\varphi }{2\pi }{ + }1} \right)T} \right] \hfill \\ \end{gathered} \right.\quad n \in N $$

$\varphi$ is the phase difference between *h*(*t*) and *g*(*t*). The WPT system operates in a resonant state. The primary side inverter full bridge power transformation function $x(t) = [x_{1} ,x_{2} ,x_{3} ,...,x_{13} ]^{{\text{T}}}$ is in phase with the power transformation function *h(t)*. According to Kirchhoff's law, the differential equation for the LCC-S WPT system:

16 $$ \left\{ \begin{gathered} L_{f} \frac{{dI_{Lf} (t)}}{dt} = g(t)V_{dc} - R_{Lf} I_{Lf} (t) - V_{cf} (t) \hfill \\ C_{f} \frac{{dV_{cf} (t)}}{dt} = I_{Lf} (t) - I_{p} (t) \hfill \\ C_{p} \frac{{dv_{cp} (t)}}{dt} = I_{p} (t) \hfill \\ L_{p} \frac{{dI_{p} (t)}}{dt} = M\frac{{dI_{s} (t)}}{dt} + V_{cf} (t) - R_{p} I_{p} (t) - V_{cp} (t) \hfill \\ L_{s} \frac{{dI_{s} (t)}}{dt} = M\frac{{dI_{p} (t)}}{dt} - V_{Cs} (t) - R_{s} I_{s} (t) - h(t)V_{c1} (t) \hfill \\ C_{s} \frac{{dv_{c1} (t)}}{dt} = I_{s} (t) \hfill \\ C_{1} \frac{{dv_{c1} (t)}}{dt} = h(t)I_{s} (t) - \frac{{V_{c1} (t)}}{{R_{L} }} \hfill \\ \end{gathered} \right. $$

The generalized state variable is:

17 $$ \left\{ \begin{gathered} \left\langle {I_{Lf} } \right\rangle_{1} = x_{1} + jx_{2} \, \left\langle {V_{Cf} } \right\rangle_{1} = x_{3} + jx_{4} \, \left\langle {V_{Cp} } \right\rangle_{1} = x_{5} + jx_{6} \hfill \\ \left\langle {I_{p} } \right\rangle_{1} = x_{7} + jx_{8} \, \left\langle {I_{s} } \right\rangle_{1} = x_{9} + jx_{10} \, \left\langle {V_{Cs} } \right\rangle_{1} = x_{11} + jx_{12} \hfill \\ \left\langle {V_{C1} } \right\rangle_{o} = x_{13} \, \hfill \\ \end{gathered} \right. $$

The generalized state space equation of the WPT system is obtained as follows:

18 $$ \left\{ \begin{gathered} x(t) = Ax(t) + Bu(t) \hfill \\ y(t) = Cx(t) + Du(t) \hfill \\ \end{gathered} \right.,\quad t \ge 0 $$

*u(t)* is the input voltage of the LCC-S compensation network, *y(t)* is the system output voltage, and the coefficient matrix in the generalized state-space equation system is:

$$ A = \left[ {\begin{array}{*{20}c} { - \frac{{R_{Lp1} }}{{L_{p1} }}} & \omega & { - \frac{1}{{L_{p1} }}} & 0 & 0 & 0 & 0 & 0 & 0 & 0 & 0 & 0 & 0 \\ { - \omega } & { - \frac{{R_{Lp1} }}{{L_{p1} }}} & 0 & { - \frac{1}{{L_{p1} }}} & 0 & 0 & 0 & 0 & 0 & 0 & 0 & 0 & 0 \\ {\frac{1}{{C_{p1} }}} & 0 & 0 & \omega & 0 & 0 & { - \frac{1}{{C_{p1} }}} & 0 & 0 & 0 & 0 & 0 & 0 \\ 0 & {\frac{1}{{C_{p1} }}} & { - \omega } & 0 & 0 & 0 & 0 & { - \frac{1}{{C_{p1} }}} & 0 & 0 & 0 & 0 & 0 \\ 0 & 0 & 0 & 0 & 0 & \omega & {\frac{1}{{C_{p1} }}} & 0 & 0 & 0 & 0 & 0 & 0 \\ 0 & 0 & 0 & 0 & { - \omega } & 0 & 0 & {\frac{1}{{C_{p1} }}} & 0 & 0 & 0 & 0 & 0 \\ 0 & 0 & {\frac{{L_{s} }}{\Delta }} & 0 & { - \frac{{L_{s} }}{\Delta }} & 0 & { - \frac{{R_{LP} L_{s} }}{\Delta }} & \omega & { - \frac{{R_{Ls} M}}{\Delta }} & 0 & { - \frac{M}{\Delta }} & 0 & 0 \\ 0 & 0 & 0 & {\frac{{L_{s} }}{\Delta }} & 0 & { - \frac{{L_{s} }}{\Delta }} & { - \omega } & { - \frac{{R_{LP} L_{s} }}{\Delta }} & 0 & { - \frac{{R_{Ls} M}}{\Delta }} & 0 & { - \frac{M}{\Delta }} & { - \frac{2M}{{\pi \Delta }}} \\ 0 & 0 & {\frac{M}{\Delta }} & 0 & { - \frac{M}{\Delta }} & 0 & { - \frac{{R_{LP} M}}{\Delta }} & 0 & { - \frac{{R_{Ls} L_{sp} }}{\Delta }} & \omega & { - \frac{{L_{p} }}{\Delta }} & 0 & 0 \\ 0 & 0 & 0 & {\frac{M}{\Delta }} & 0 & { - \frac{M}{\Delta }} & 0 & { - \frac{{R_{LP} M}}{\Delta }} & { - \omega } & { - \frac{{R_{Ls} L_{p} }}{\Delta }} & 0 & { - \frac{{L_{p} }}{\Delta }} & {\frac{{2L_{p} }}{\pi \Delta }} \\ 0 & 0 & 0 & 0 & 0 & 0 & 0 & 0 & {\frac{1}{{C_{s} }}} & 0 & 0 & \omega & 0 \\ 0 & 0 & 0 & 0 & 0 & 0 & 0 & 0 & 0 & {\frac{1}{{C_{s} }}} & { - \omega } & 0 & 0 \\ 0 & 0 & 0 & 0 & 0 & 0 & 0 & 0 & 0 & { - \frac{4}{{\pi C_{o} }}} & 0 & 0 & { - \frac{1}{{R_{L} C_{o} }}} \\ \end{array} } \right] $$

$$ B = [\begin{array}{*{20}c} 0 & { - \frac{2}{{\pi L_{p1} }}} & 0 & 0 & 0 & 0 & 0 & 0 & 0 & 0 & 0 & 0 & 0 \\ \end{array} ]^{T} $$

$$ C = [\begin{array}{*{20}c} 0 & 0 & 0 & 0 & 0 & 0 & 0 & 0 & 0 & 0 & 0 & 0 & 1 \\ \end{array} ] $$

$$ D = [0] $$

$\Delta = M^{2} - L_{p} L_{s}$, adding disturbances to the state variables and input variables of the WPT system, it can be concluded that:

19 $$ \left\{ \begin{gathered} x(t) = X + \hat{x}(t) \hfill \\ u(t) = U + \hat{u}(t) \hfill \\ y(t) = Y + \hat{y}(t) \hfill \\ \end{gathered} \right. $$

*X, U, Y* is the steady-state value of each state variable in the WPT system, and the expression for the steady-state value can be obtained when the state variable does not change over time:

20 $$ \left\{ \begin{gathered} X = - A^{ - 1} BU \hfill \\ Y = CX + DU \hfill \\ \end{gathered} \right. $$

By substituting Eq. (20) into the generalized state-space expression (19) of the WPT system, the generalized state-space equation of WPT system small-signal modelling based on the LCC-S compensation network can be obtained:

21 $$ \left\{ \begin{gathered} \dot{\hat{x}}(t) = A\hat{x}(t) + B\hat{u}(t) \hfill \\ \hat{y}(t) = C\hat{x}(t) + D\hat{u}(t) \hfill \\ \end{gathered} \right. $$

The coefficient matrix of Eq. (21) is the same as of Eq. (18), and the open-loop small-signal modelling transfer function of the WPT system based on the LCC-S compensation network can be obtained:

22 $$ G_{LCC - S} (s) = C(sI - A)^{ - 1} B + D $$

**Figure 10:**
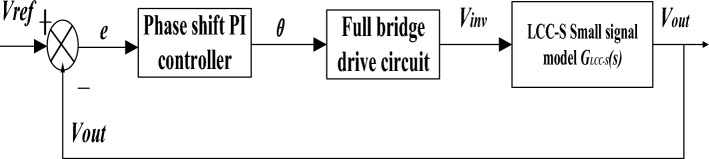
PI closed-loop control block diagram of the WPT system under the phase-shifting operation mode.

The closed-loop control block diagram of the WPT system based on the LCC-S compensation network is shown in Fig. 10. The difference between the reference output voltage and the actual output voltage enters the phase-shifting PI controller. The input of the small-signal modelling of LCC-S established by Eq. (22) is the input voltage of the inverter circuit, and the variable output by the phase-shifting PI controller is the input voltage of the inverter. The control quantity is converted into the phase shift angle of the two bridge arm driving signals of the full bridge inverter circuit through Eq. (23). It enters the inverter power supply driving circuit to control the output voltage of the inverter power supply and complete the control of the secondary side output voltage.

23 $$ \theta = \frac{360}{\pi }\sin^{ - 1} \left( {\frac{{V_{dc} }}{{V_{in} }}} \right) $$

### Simulation analysis of LCC-S multi-transmitter wireless power transfer system

**Figure 11:**
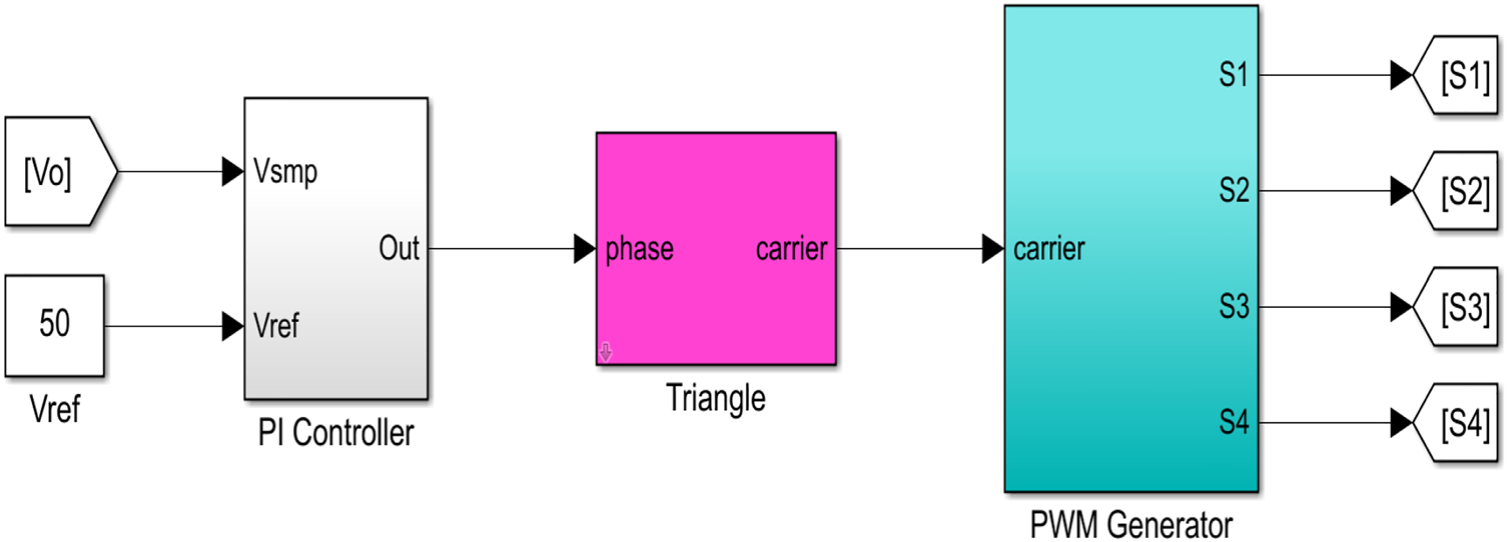
PI control of array multi-transmitter WPT system.

The PI control module of the 4-TX coils WPT system based on the LCC-S compensation network in Fig. 11. Preset the output voltage value for the WPT system, set it to 50 V, and send the difference between the output voltage and the preset voltage value to the PI regulator. Use the triangular wave as the carrier, and compare the output voltage value after PI adjustment with the triangular wave to generate the corresponding PWM wave after phase shift angle change and input it into the full bridge inverter circuit. Control the phase shift angle of the two bridge arms in the full bridge inverter circuit to achieve the WPT system output at the preset voltage value. Import the open-loop small signal transfer function of the WPT system shown in Eq. (23) into the PID toolbox of MATLAB, and obtain the appropriate PI controller parameters: *K*_p_ = 0.007,* K*_i_ = 5. The parameters of the resonance compensation network are shown in Table 1.

**Table 1:** WPT system parameters.

System parameter	Parameter value
Primary/secondary side coil self-inductance $L_{S} /L_{R}$	102.48 μH
Coupling coefficient $k$	0.15–0.30
Primary side compensation inductance $L_{S1} /L_{R2}$	45.18 μH
Primary side parallel compensation capacitor $C_{S1} /C_{R2}$	77.932 nF
Primary side series compensation capacitor $C_{1} /C_{2}$	60.994 nF
Secondary measurement series compensation capacitor $C_{s}$	34.211 nF
DC input voltage $U_{DC}$	100 V

**Figure 12:**
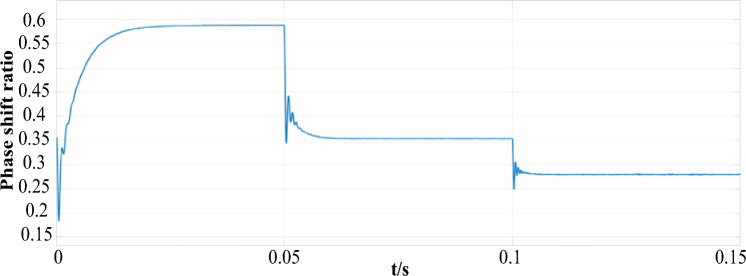
Comparison of multi-mode downshift of array multi-launch WPT system.

The multi-transmitter WPT system is set to switch from a single transmitter to 2-TX mode and from 2-TX coil mode transfer to 4-TX mode. Figure 11 shows the multi-transmitter WPT system multimodal phase shift ratio curve during operation. It can be seen from Fig. 12 that the phase-shifting ratio of the 1-TX coil mode is 0.5878, the phase-shifting ratio of the 2-TX coil mode is 0.3535, and the phase-shifting ratio of the 4-TX coil mode is 0.2807.

**Figure 13:**
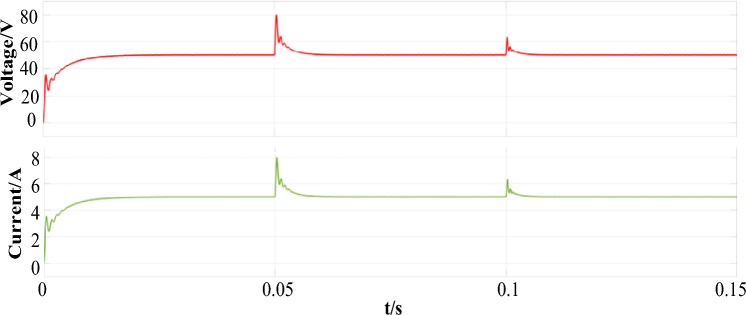
Load output current and voltage.

The load output waveform of the array multi-transmitter WPT system is shown in Fig. 13. The current and voltage waveform of the load resistor in the WPT system undergoes switching at *t* = 0.05 s, *t* = 0.1 s. After a short recovery time, the system outputs 50 V in all three operating modes. Due to the pure resistance of 10 Ω in the load, the current output waveform is 5 A.

### Experimental verification of multimodal constant voltage output

**Figure 14:**
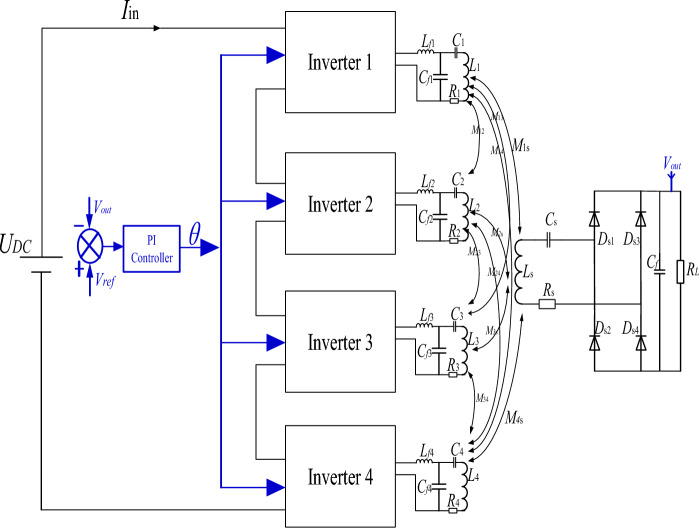
Multi-mode switching control block diagram of the multi-transmitter WPT system.

In the control block diagram of the multi-transmitter WPT system in Fig. 14, the PI closed-loop control section is in blue. The system load voltage value is returned to the transmitter controller through a voltage sensor, and the measured voltage value is compared with the reference output voltage value. The error transmits to the PI controller to calculate the phase shift angle of the inverter circuit driving signal. By switching the working modes of the WPT system through a multi-transmitter switching control system, the measured load voltage values under different working modes of the WPT system are compared with the reference output voltage. After passing through the PI controller at the transmitter, the inverter circuit driving signal with phase shift angle is generated and transmitted to the corresponding full bridge circuit under different working modes of the transmitters, thereby maintaining the constant voltage output of the multi-transmitter WPT system under different work modes.

**Figure 15:**
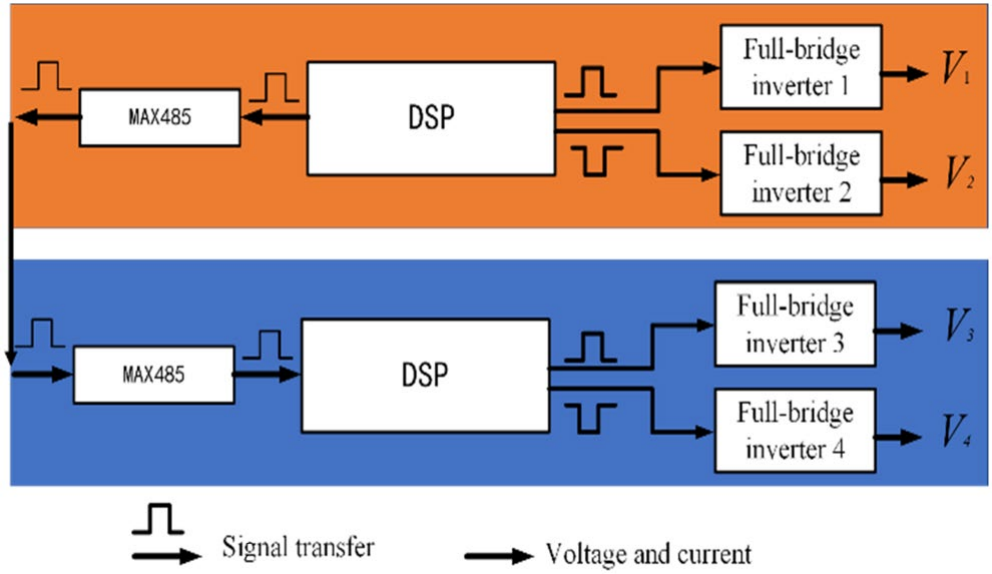
Transfer circuit diagram of the drive signal based on master–slave DSP.

The output current of multiple inverters is not synchronized. The magnetic field generated by the corresponding transmitter will partially offset and reduce the transfer efficiency of the system. Therefore, it is necessary to take measures to realize the synchronous output of multiple inverter channels. Therefore, this paper designs a high-frequency inverter power supply based on dual DSP master–slave synchronization architecture. DSP1 is the central inverter unit, and DSP2 is the square wave driving signal from the inverter unit to the central inverter unit. After receiving the drive signal from the inverter unit DSP2, an appropriate delay compensation is required to send the square wave drive signal, as shown in Fig. 15. The DSP needs to detect the rising edge and falling edge of the driving waveform, calculate the output waveform after adding phase angle compensation, and finally generate the lower tube drive. MAX485 is a communication module that sends and receives communication signals. Assuming that the phase of the delay is $\theta_{delay}$, the clock frequency of the DSP series is $f_{DSP}$, and the number of clock cycles required by the DSP to compensate for the delay is:

24 $$ T_{delay\_CLK} = \frac{{\theta_{delay} }}{360} \times \frac{{f_{DSP} }}{f} = \frac{{T_{delay} }}{T} \times \frac{1}{360} \times \frac{{f_{DSP} }}{f} = \frac{{T_{delay} }}{360} \times f_{DSP} $$

To compensate for this delay, the DSP needs a delay after detecting edge jumps:

25 $$ T_{delay\_comp\_CLK} = T_{CLK} - T_{delay\_CLK} $$

**Figure 16:**
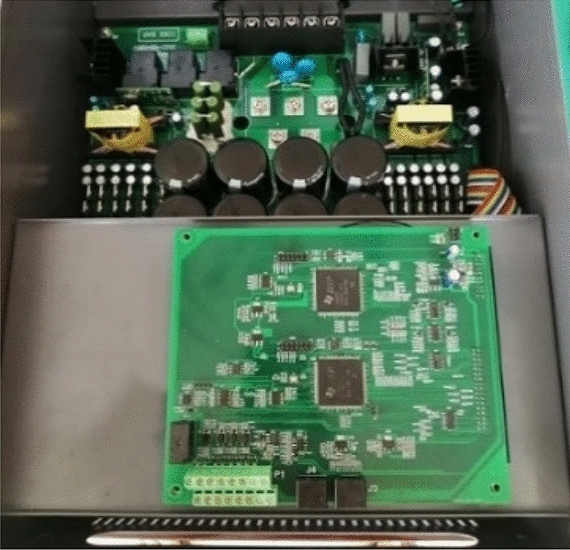
Four-channel inverter principal prototype.

The prototype of a multi-channel independent inverter power supply is shown in Fig. 16. The MOSFET adopts UJ4SC075011K4S from United SiC, and the driving chip adopts Infineon 1ED38 × 0Mc12M. The upper tube drive signal generated by the DSP of the central inverter is transmitted to the DSP of the slave inverter through the MAX485 transceiver. After the DSP of the slave inverter detects the edge jump change of the drive signal, it starts counting, and the counting value is calculated according to Eq. (25).

To verify that the multi-transmitter wireless power transfer system can realize constant output in all modes. By controlling the receiver movement to the middle position of the 4-TX coils through a rocker arm mechanism, the load current and voltage waveforms of the load are measured under three different working modes. The primary side of the WPT system includes a high-frequency inverter power supply, an LCC compensation network, and an array transmitter coil. The size of the transmitter/receiver coils is 300 mm × 300 mm. WPT system compensation network parameters are shown in Table 1.

**Figure 17:**
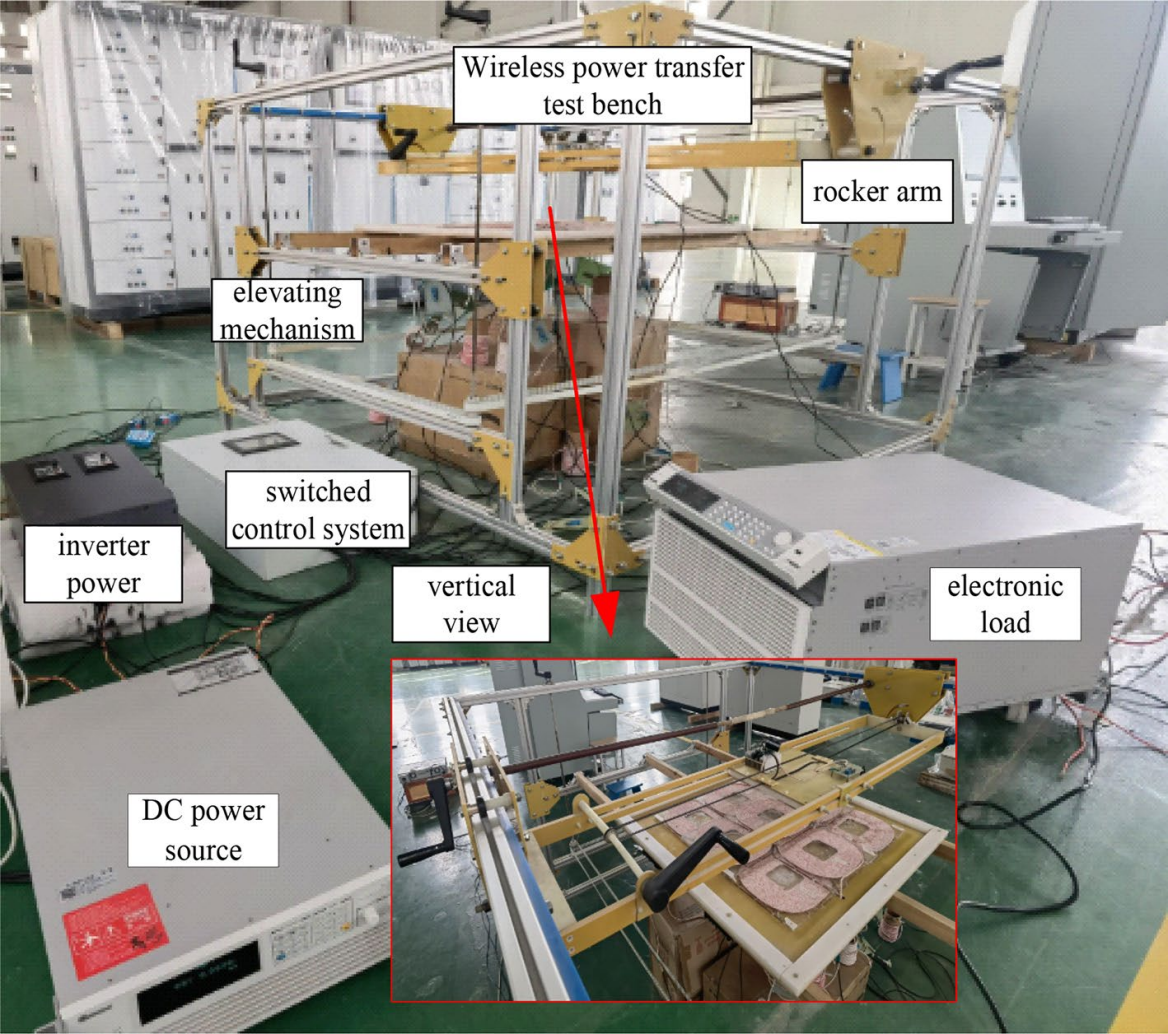
Experimental diagram of multi-mode wireless power transfer system.

**Figure 18:**
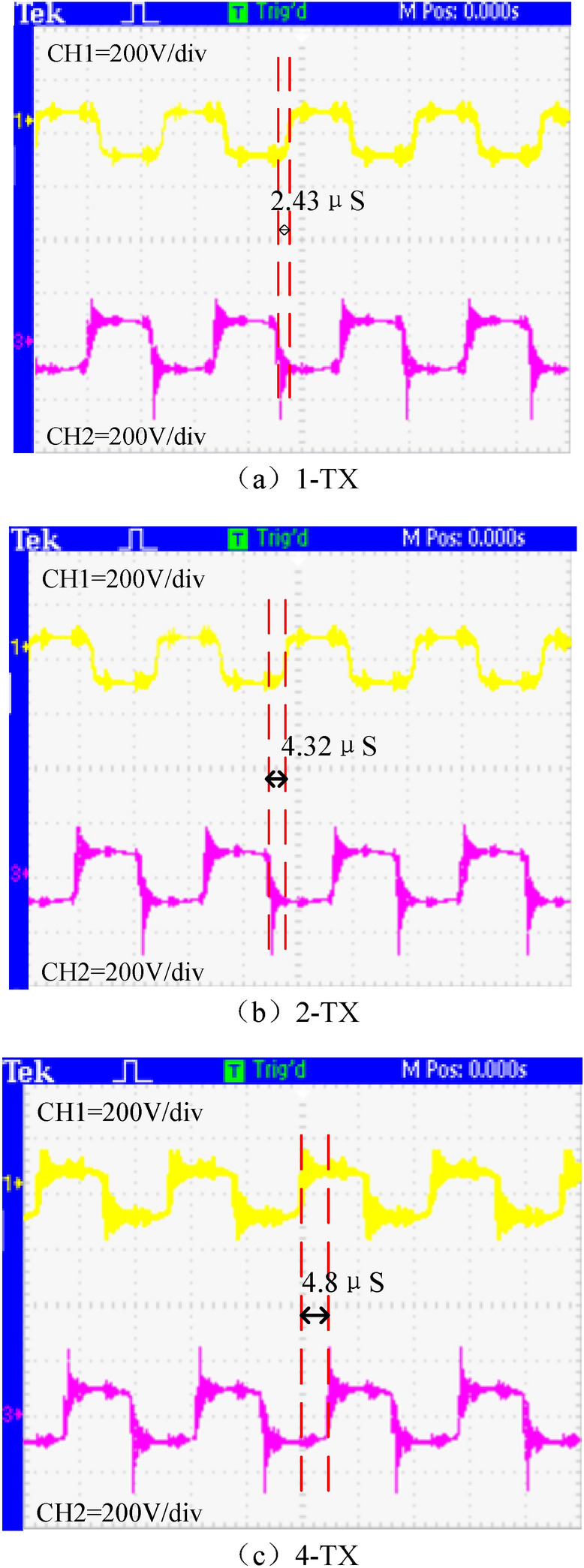
Wave of multi-mode wireless power transfer system.

The experimental prototype of the array wireless power transfer system is shown in Fig. 17.Switch the 1-TX, 2-TX, and 4-TX of the WPT system through the switching control system. The working frequency of the WPT system in this article is 85 kHz. Due to the difference between the experimental device and the simulation parameters, *K*_p_ = 0.012,* K*_i_ = 5.4 during the experiment. Measure and obtain the output voltage waveforms of the two bridge arms in the full bridge inverter circuit, and the other two voltage output waveforms complement these two voltage output waveforms. According to the inverter output voltage under the three operating modes in Fig. 18, the conduction angle of the output voltage between the two bridge arms has changed under the three transmitter modes. In the 1-TX coil, the difference in the conduction angle of the two output voltage waveforms is 2.43 μs. The conduction angle difference between the two output voltages in the 2-TX coil mode is 4.32 μs. The difference in conduction angle between the two output voltages in the 4-TX coil mode is 4.8 μs.

**Figure 19:**
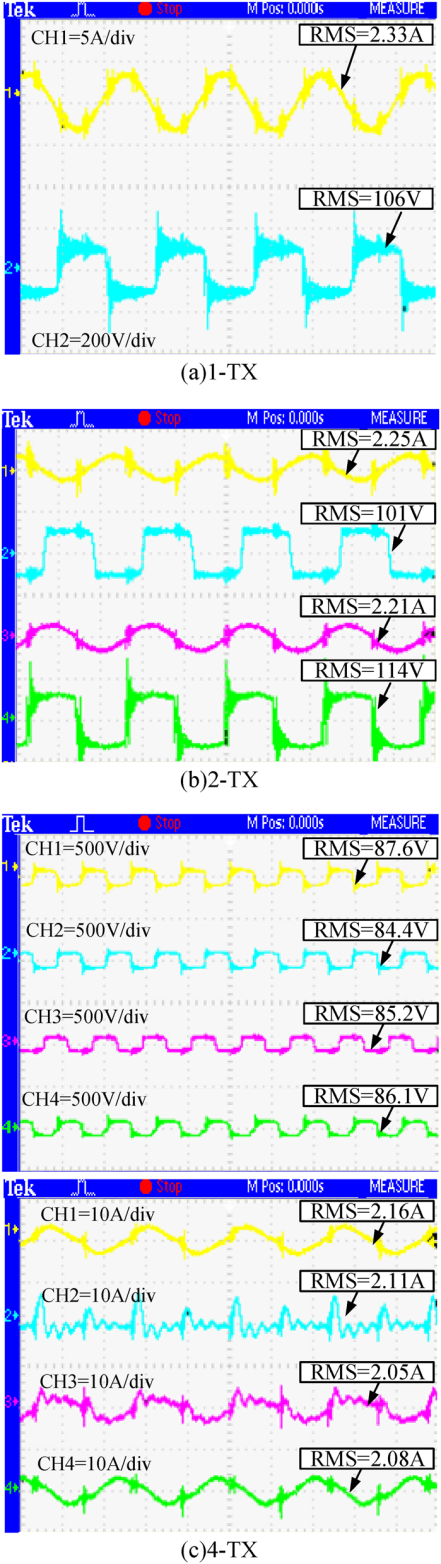
Inverter output current and voltage under three operating modes.

The output current and voltage waveforms of the inverter under the three working modes of the transmitter coil are shown in Fig. 19. The output current and voltage of the inverter in the 1-TX coil mode are the same as the output current and voltage of the inverter in the 2-TX coil. The current difference between the 2-TX coils in the 2-TX coil mode is 0.04A, and the 4-TX output current is slightly different. The maximum difference between the output channels is 0.15A. Due to the manual winding of the coil and the difference in device parameters between the 2-TX compensation networks, there is a slight difference in the inverter output current between the 2-TX coils, but it is within the allowable error range.

**Figure 20:**
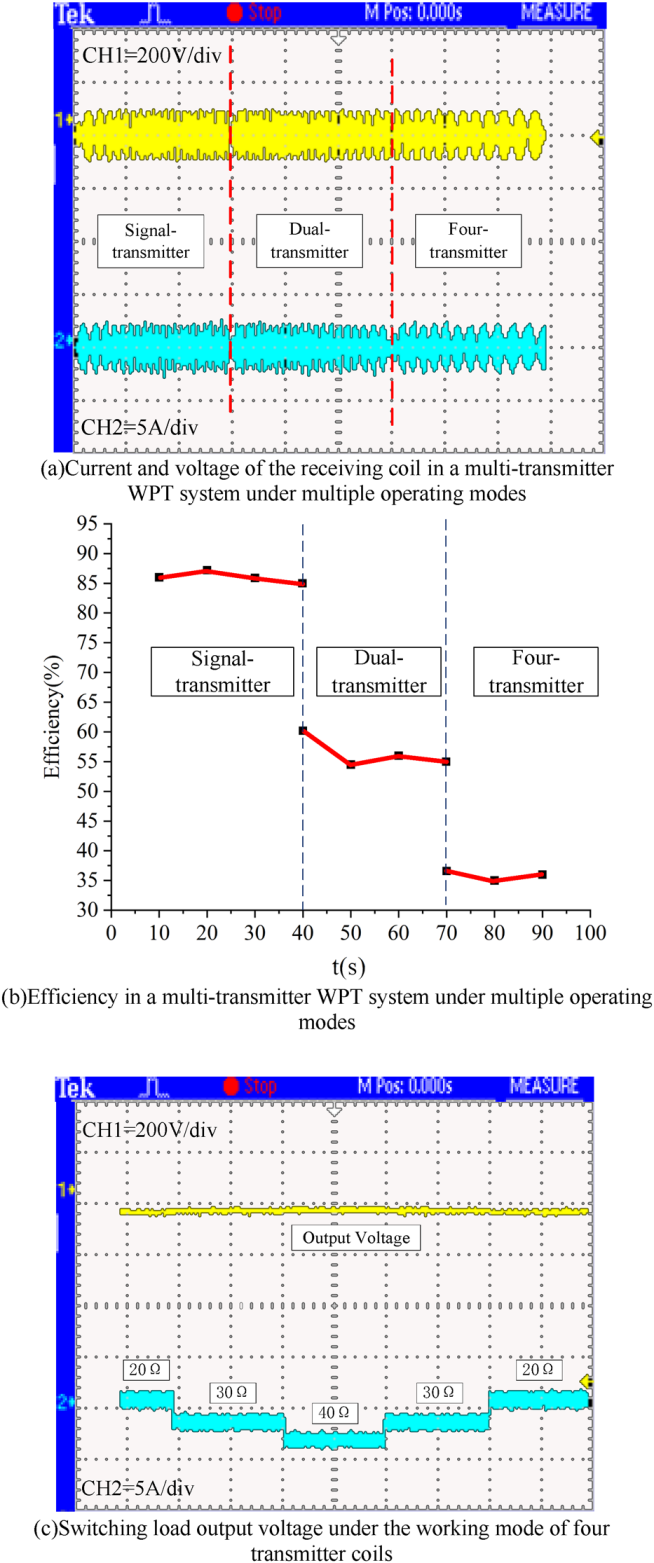
Output voltage and current.

Figure 20a shows the current and voltage of the receiver coil under switching three operating modes of the transmitter coil, which can remain stable under the three operating modes of the transmitter coil. Figure 20b shows the transfer efficiency variation curve of the WPT system under different operating modes. When the WPT system switches to the dual transmitter coil and four transmitter coil working modes, the transfer efficiency of the WPT system decreases due to the newly added area of the transmitter coils in the dual transmitter coil and four transmitter coils not facing the receiver coil. However, the transmitter coil working modes of the three WPT systems can all meet the power supply requirements of the WPT system. Figure 20c shows the current and voltage waveforms of the load under the switching resistance. Under the WPT system loads of 20 Ω, 30 Ω, and 40 Ω, the output voltage of the system can maintain a constant output. The resistance switching of the WPT system is through the electronic load Chroma 63210 (Table 2).

**Table 2:** Comparative analysis of UAV WPT system.

Reference	Coil structure	Operation mode
^16,17^	Single transmitter coils	Signal transmit
^18^	Multi-transmitter coils	Signal transmit
^19^	Capacitor plate	Signal transmit
^20^	Single transmitter coils	Battery free operation
This paper	Multi-transmitter coils	Joint operation of multitransmitter coils

## Conclusion

This paper proposes a multi-transmitter coil working mode cooperative working mechanism for an array multi-transmitter wireless power transfer system. The multi-mode output performance of the array multi-transmitter WPT system is modelled and analyzed. By adjusting the phase shift angle of the driving signal between the two branches in the full-bridge inverter circuit, the multi-transmitter WPT system based on the LCC-S compensation network can achieve stable voltage output in three different operating modes. This control method not only realizes the joint modulation of multiple transmitters and different working modes in the multi-transmitter WPT system but also enhances the redundancy and power robustness of the multi-transmitter WPT system. It is beneficial for improving the robustness of the unmanned aerial vehicle WPT system.

## Data Availability

The datasets used and/or analysed during the current study available from the corresponding author on reasonable request.
